# Scent of stem cells: How can neurogenesis make us smell better?

**DOI:** 10.3389/fnins.2022.964395

**Published:** 2022-08-03

**Authors:** Vittoria Avaro, Thomas Hummel, Federico Calegari

**Affiliations:** ^1^Centre for Regenerative Therapies Dresden (CRTD), Technische Universität Dresden, Dresden, Germany; ^2^Department of Otorhinolaryngology, Smell and Taste Clinic, Technische Universität Dresden, Dresden, Germany

**Keywords:** adult neurogenesis, olfactory epithelium, olfactory bulb, olfaction, odor sensitivity, odor discrimination

## Abstract

Throughout the animal kingdom, olfaction underlies the ability to perceive chemicals in the environment as a fundamental adaptation with a plethora of functions. Unique among senses, olfaction is characterized by the integration of adult born neurons at the level of both the peripheral and central nervous systems. In fact, over the course of life, Neural Stem Cells (NSCs) reside within the peripheral Olfactory Epithelium (OE) and the brain’s subventricular zone that generate Olfactory Sensory Neurons (OSNs) and interneurons of the Olfactory Bulb (OB), respectively. Despite this unique hallmark, the role(s) of adult neurogenesis in olfactory function remains elusive. Notably, while the molecular signature and lineage of both peripheral and central NSC are being described with increasing detail and resolution, conflicting evidence about the role of adult born neurons in olfactory sensitivity, discrimination and memory remains. With a currently increasing prevalence in olfactory dysfunctions due to aging populations and infections such as COVID-19, these limited and partly controversial reports highlight the need of a better understanding and more systematic study of this fascinating sensory system. Specifically, here we will address three fundamental questions: What is the role of peripheral adult neurogenesis in sustaining olfactory sensitivity? How can newborn neurons in the brain promote olfactory discrimination and/or memory? And what can we learn from fundamental studies on the biology of olfaction that can be used in the clinical treatment of olfactory dysfunctions?

## Introduction

As part of the nervous system, our senses are highly plastic. They adapt to our experiences and the world that surrounds us ultimately influencing how we experience it. Accordingly, sensory loss often evokes remodeling and adaptation of our other senses improving or reallocating their function (Collignon et al., 2009; Sathian and Stilla, 2010; Frasnelli et al., 2011). Among the five senses, olfaction additionally possesses a unique hallmark giving it increased potential for plasticity: The persistent addition of newborn neurons over the course of life both at the level of the peripheral and central nervous system.

In vertebrates, odor detection begins when odorants reach the Olfactory Epithelium (OE), part of the peripheral nervous system. Here, Olfactory Sensory Neurons (OSNs) express G-coupled Olfactory Receptors (ORs) encoded by more than 1,000 genes in rodents and nearly 400 in humans making them the largest gene family in our genomes (Buck and Axel, 1991; Glusman et al., 2001; Young et al., 2002). To date, the expression of ORs is considered to be stochastic and regulated by both chromatin remodeling and enhancer activity (reviewed in Pourmorady and Lomvardas, 2022). While newborn, immature neurons transiently co-express different receptors, their maturation ultimately restricts their fate into the expression of only one OR defining OSN identity throughout its lifespan (Pourmorady and Lomvardas, 2022). This expression of one OR with its own affinity for different molecules, together with the relative abundance of different types of OSNs expressing different ORs, defines the sensitivity of the whole OE. Interestingly, recurrent odor stimulation was reported to change the abundance of responsive OSNs (Ibarra-Soria et al., 2017) indicating that olfactory experience can modulate sensitivity. However, the mechanisms underlying this type of plasticity are currently not known.

Upon activation, OSNs initiate action potentials encoding and transmitting information to the brain’s Olfactory Bulb (OB), the first olfactory station of the central nervous system. Here, projections of OSNs form synapses with mitral and tufted cells within spherical structures, nearly 2,000 in mouse and 6,000 in human, known as glomeruli (Maresh et al., 2008). Notably, axons of OSNs expressing the same receptor will converge to the same glomerulus, and make synapses with the same set of projection neurons, namely Mitral and Tufted cells (M/T cells). Therefore, any scent composed of a mix of monomolecular odorants will activate different sets of OSNs in the OE corresponding to the activation of a specific set of glomeruli in the OB and resulting in an anatomical and topographic map of the smell perceived (Mombaerts et al., 1996; Mizrahi, 2018; Lodovichi, 2021). In this context, it is worth mentioning that the functional significance of a change in the ratio of OR genes and the number of glomeruli (ca. 1:2 in mouse and 1:16 in humans) is not known.

In addition to the topographic organization of glomeruli, another level of control is imposed by local and centrifugal inhibitory circuits in which OB interneurons are key players (Burton, 2017). The most abundant population of GABA interneurons in the OB is constituted by Granule Cells (GCs), present in the inner layer of the OB. Extending their apical dendrites, GCs form lateral dendrodentritic synapses with 200–300 M/T cells simultaneously. Each activated M/T cell releases glutamate in that synapsis, activating GCs and, thus, self-inhibiting itself and all the other M/T cells connected to the same GC (Burton, 2017). In concert, GCs receive robust glutamatergic inputs from the so-called Olfactory Cortex (OC), an ensemble of cortical structures that process olfactory signals. Glutamatergic neurons in the piriform cortex as well as other cortical regions such as the anterior olfactory nucleus send long-range centrifugal projections back to the OB controlling GCs and, consequently, M/T cells activity (Boyd et al., 2012; Markopoulos et al., 2012; Burton, 2017). Olfactory inputs in the OB are processed as a result of both the topographic organization of glomeruli and of inhibitory circuits regulated by interneurons. Supported by both, OB activity underlies our ability to discriminate between different odorants. Once processed in the OB, olfactory signals are transmitted to the OC where they are thought to be stored as memory integrated through a complex wiring system to other brain regions (Meissner-Bernard et al., 2019).

In summary, fundamental features characterizing the olfactory system, namely sensitivity, discrimination, and memory can overall be assigned to the three regions of the OE, OB, and OC, respectively. In turn, this raises the question: Can adult neurogenesis modulate any of these three fundamental abilities?

Specifically, both the OE and the OB are characterized by the integration of newborn neurons throughout life. Within the OE, Neural Stem Cells (NSCs) were first described over four decades ago (Graziadei and Graziadei, 1979) but only very recently characterized at the molecular and cellular level by single-cell RNA sequencing in rodents (Fletcher et al., 2017) and humans (Durante et al., 2020). At the level of the central nervous system, while partly debated in humans (Curtis et al., 2007; Sanai et al., 2011; Lötsch et al., 2014; Paredes et al., 2016), NSCs residing within the sub-ventricular zone generate newborn neurons migrating through the rostral migratory stream and integrating in the OB (Doetsch et al., 1997). Here, most newborn neurons integrate as GCs (Alvarez-Buylla and García-Verdugo, 2002), already mentioned above, while a small population differentiate in periglomerular cells, interneurons regulating glomerular activity by synapsis formation with both M/T cells and OSNs axons (reviewed in Wu et al., 2020; Capsoni et al., 2021).

While NSCs within both the OE and OB have long been considered important to maintain structural plasticity underlying homeostasis and regeneration, several fundamental questions arise with regard to additional roles of adult neurogenesis in odor sensitivity, discrimination, and/or memory. Here, we will address central questions pertaining to the role of newborn neurons in olfaction, namely: (i) Can peripheral adult neurogenesis in the OE promote odor sensitivity? (ii) Are newborn neurons in the OB promoting discrimination, memory, or both? And (iii) how can studies in model organisms help us define novel treatments for olfactory dysfunction in a clinical setting?

## Can adult neurogenesis in the olfactory epithelium promote odor sensitivity?

The OE is the most active neurogenic niche and the only one characterized in a peripheral sensory system (Brann and Firestein, 2014). Due to their constant exposure to the environment, OSNs are highly vulnerable and their lifespan short ranging from 1 to 3 months (Hinds et al., 1984; Kondo et al., 2010; McClintock et al., 2020). This feature primarily led to the concept that adult neurogenesis in the OE exclusively serves tissue homeostasis and regeneration. Little attention, however, was paid to additional potential roles in promoting olfactory sensitivity.

Olfactory sensitivity is defined by the lowest concentration of an odorant that can be detected. Since most ORs are promiscuous and bind different odorants with different affinity, it is intuitive to conclude that OE sensitivity may ultimately depend on the number of OSNs expressing a given OR, the relative proportion of OSNs expressing it as well as the expression level of a given OR in a particular set of OSNs. Notably, adult neurogenesis inherently provides the means to constantly renew the number of OSNs and the OR expression pattern of the whole OE, hence, dynamically remodeling its sensitivity. However, to date this seemingly logical inference was not experimentally validated and the molecular mechanisms underlying it not investigated.

**FIGURE 1:**
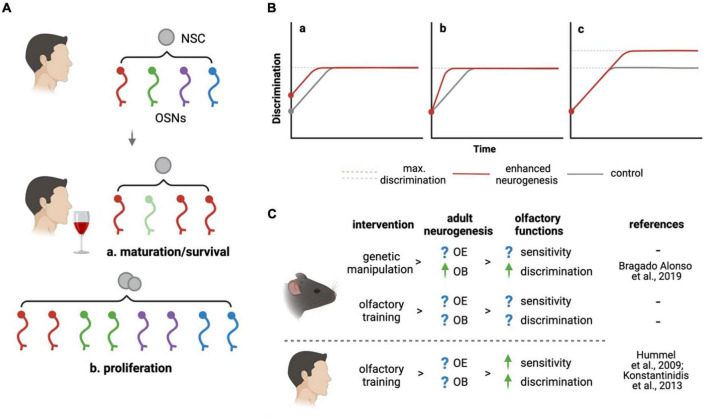
How can adult neurogenesis promote olfaction? **(A)** At the level of the peripheral nervous system, adult neurogenesis may respond to olfactory stimuli changing the abundance of specific OSNs (indicated by colors) by (a) changing the maturation or survival of immature OSNs or (b) increasing neurogenesis itself. **(B)** Effects of enhanced (red lines) relative to basal (gray) brain neurogenesis on olfactory discrimination are modeled based on (a) improved innate ability, (b) learning, or (c) maximal performance. Combinations thereof were not depicted. **(C)** Olfactory experience may act both in humans and rodents on olfactory neurogenic niches supporting functions such as sensitivity or discrimination. Converse to Bragado Alonso et al. (2019) decrease in neurogenesis and discrimination was reported by Li et al. (2018). Figure created with BioRender.

Here, we envision different models by which adult neurogenesis may drive the remodeling of olfactory sensitivity under olfactory experiences such as increased stimulation of the OE or, conversely, odorant deprivation or desensitization. In addition to a change in the expression levels of a given OR within mature OSNs, it is important to consider that olfactory experience may influence the choice of OR expression itself within immature, newborn neurons (Kim et al., 2020). This, in turn, may promote the integration and survival of such newborn OSNs upon their targeting of glomeruli in the OB that are dedicated to the processing of such odorants. By guiding OR choice as well as maturation and/or survival of newborn OSNs, adult neurogenesis may therefore adapt odor sensitivity to novel environmental stimuli through a change in the number of specific OSNs (Figure 1A).

While this effect of olfactory experience in regulating the specification and survival of newborn neurons would seem intuitive, to our knowledge no report characterized it thus far. In addition, an even more intriguing possibility is that olfactory experience may itself regulate the activity of the NSCs themselves. Increased levels of adult neurogenesis would, in turn, provide increased numbers of OSNs whose OR expression may be additionally regulated either through stimuli-dependent maturation, as described above, or stochastically, to adapt sensitivity to novel stimuli (Figure 1A). Such links between environmental stimuli, NSC activity, neuronal specification and survival were only partly described in the OE (Watt et al., 2004) but well documented in other neurogenic niches, most notably the hippocampus (Van Praag et al., 1999; Kempermann, 2019) and consistent with the notion that traits providing synergistic advantages are evolutionary conserved.

## Are newborn neurons in the olfactory bulb promoting discrimination, memory, or both?

Unlike the OE, the cellular composition and physiology of the central station of the OB are well characterized. Yet, the role of adult neurogenesis in the OB has remained elusive. In the last years, first studies have suggested a role of newborn neurons in supporting olfactory memory. Partly contradicting each other, these studies suggested effects of adult neurogenesis on short-term (Breton-Provencher et al., 2009) or long-term (Lazarini et al., 2009; Sultan et al., 2010) memory that later reports did not corroborate. In parallel, support for a role of adult OB OB neurogenesis in olfactory discrimination has grown (discussed below). In this context, it should be reminded that the primary neuronal population of the OB, the GCs that are constantly renewed by adult neurogenesis (Imayoshi et al., 2008), are fundamental in establishing both local and centrifugal inhibitory circuits known to promote sparseness in mitral and tufted cells activity (Gschwend et al., 2015; Wu et al., 2020). More thoroughly studied in the context of hippocampal function, sparseness minimizes the overlap between patterns of neuronal activity, which is thought to be key in pattern separation. Therefore, by analogy, it would be expected that an increase in adult neurogenesis and abundance of inhibitory GCs would promote sparseness, hence, discrimination between similar odorants. On the other hand, periglomerular interneurons are uniglomerular and not involved in lateral inhibition thought to be crucial in supporting OB’s pattern separation and therefore discrimination (Wu et al., 2020).

Experimental evidences were provided in support of the above-mentioned hypothesis. Inhibiting adult neurogenesis resulted in impaired discrimination (Li et al., 2018) while, conversely, a genetic increase in NSC expansion and neurogenesis improved it (Bragado Alonso et al., 2019). Notably, in both studies changes in olfactory performance became evident only when mice were subjected to complex discrimination tasks using highly similar odorants. The fundamental question arising is whether adult neurogenesis improved the animal’s innate ability to better discriminate similar odorants or, rather, the learning process leading to their discrimination. Specifically, in Figure 1B we graphically depict all possibilities that, on purely theoretical grounds, are possible to explain how increased neurogenesis may provide the OB with (a) an innate, intrinsic ability to better discriminate independently from learning, (b) a faster learning process while reaching the same maximum discrimination performance, (c) an enhanced discrimination performance upon learning is completed, or (d) any combination thereof (not depicted). While all these possibilities were not systematically assessed, the two above mentioned studies upon ablation or enhancement of neurogenesis allow us to restrict the 9 possible permutations of 3 possibilities to at least reject 1, and validate 1, of these effects.

Specifically, excluding the first model discussed (a), Li et al. (2018) showed that ablation of OB neurogenesis did not affect the mice intrinsic ability to discriminate, which was similar at the start of complex discrimination tasks. In addition, and confirming at least the third model discussed (c), Bragado Alonso et al. (2019) showed that a converse increase in OB neurogenesis improved the mice performance over the baseline after learning was complete. However, neither of the two study confirm, or exclude, the effects of the second model discussed on the speed of the learning process itself (b). Notably, Alonso et al. (2012) reported that the artificial activation of adult-born neurons in the OB facilitates learning only in fine discrimination task. Many other questions remain. What are the molecular mechanisms underlying the effects of olfactory stimuli on adult neurogenesis and, reciprocally, how can adult neurogenesis modulate olfactory experience? Above all, can principles emerging from fundamental studies have an impact on the clinical treatment of olfactory dysfunction?

## Can studies in model organisms help define novel treatments of olfactory dysfunction?

For obvious reasons, adult human neurogenesis in the central nervous system is poorly characterized and long debated both at the level of the hippocampus (Kempermann et al., 2018; Sorrells et al., 2018) and the human sub ventricular zone (Curtis et al., 2007; Sanai et al., 2011). On the other hand, at the peripheral level, the existence of an active pool of NSCs during adulthood in humans is formally accepted (Durante et al., 2020). While new evidence supporting adult neurogenesis in the human brain continue to emerge (Wang et al., 2022; Zhou et al., 2022), we will nonetheless restrict our discussion to the peripheral nervous system. In particular, NSCs in the human OE represents a particularly appealing target of intervention due to their easy accessibility. Even more so, and as recently emphasized by the ongoing COVID-19 pandemic, infections of the OE represent one of the most prevailing causes of olfactory dysfunction. Can controlled olfactory stimulation be used as a means to promote the recovery of olfactory functions? And can direct manipulation of NSCs be used to harness their potential to treat smell disorders?

Ongoing clinical practice provides a remarkable example of how olfactory experience may trigger a recovery in olfactory functions. Pioneered by the Hummel group (Hummel et al., 2009), olfactory training has become a well-established treatment consisting of repetitive short-term exposures to a specific set of odorants [rose (phenyl ethyl alcohol), clove (eugenol), eucalyptus (eucalyptol), lemon (citronellal)]. By this, patients are exposed to 4 different odorants over a period of 4 months, after which, between 30 and 60% of the patients recover their sense of smell, which, notably, is independent from the etiology of the dysfunction (Hummel et al., 2009; Konstantinidis et al., 2013). Remarkably, in this area clinical practice has anticipated biological research meaning that mechanisms underlying the efficacy of this treatment are still completely unknown. Can activation of NSC and enhanced adult neurogenesis be the key mechanism behind olfactory training? Can failure in the activation of NSC and neurogenesis explain the remaining 40–70% of cases in which olfactory training remains ineffective? Indirect evidence suggests that these possibilities are likely.

First, olfactory training was found to be more effective in younger patients (Patel et al., 2017; Nguyen and Patel, 2018; Saatci et al., 2020) and, second, less effective in post-traumatic olfactory loss associated with damage of the brain, rather than the OE (Konstantinidis et al., 2013; Poletti et al., 2017). In addition, third, the efficacy of this treatment is largely independent on the specific set of odorants used during training and equally effective in recovery of sensitivity to a broader set of odorants (Altundag et al., 2015; Croy et al., 2015; Oleszkiewicz et al., 2021). Together, this evidence is consistent with a model thereby repetitive and chronic exposure to strong odorants during olfactory training may trigger activation of NSC and/or, as discussed above, increase the integration and survival of newborn neurons as well as modulate their expression of ORs. If validated, these mechanisms may provide new means of clinical intervention in the treatment of olfactory dysfunctions that are currently more pervasive than ever before.

## Conclusion and future outlook

Here, we discussed how adult olfactory neurogenesis not only sustains the structural and cellular homeostasis of the olfactory system but also how it may actively promote its functionality based on environmental stimuli at both the peripheral and central levels. In parallel, we formulated several questions pertaining to the mechanisms underlying such effects and proposed hypotheses to explain how neurogenesis may be critical in understanding the fundamental biology of the sense of smell, and improving the clinical treatment of olfactory dysfunction (Figure 1C). While experimental validations and characterizations of these hypotheses are missing, we hope that our contribution may help in highlighting the need of more concerted efforts between fundamental research and clinical studies in a better understanding of this fascinating function evolutionary conserved across the animal kingdom.

## Data availability statement

The original contributions presented in this study are included in the article/supplementary material, further inquiries can be directed to the corresponding author/s.

## Author contributions

VA and FC conceived and wrote the manuscript. TH revised the manuscript. All authors approved the submitted version.
